# The Dynamics of Human Cytomegalovirus Markers in Tuberculosis Disease Among People With Human Immunodeficiency Virus on Long-term Antiretroviral Therapy: A Nested Case-Control Study

**DOI:** 10.1093/ofid/ofag015

**Published:** 2026-01-14

**Authors:** Sivaporn Gatechompol, Sasiwimol Ubolyam, Thitiporn Somjit, Sasitorn Plakunmonthon, Chavachol Setthaudom, Anchalee Avihingsanon, Stephen J Kerr, Frank van Leth, Frank Cobelens

**Affiliations:** Division of Infectious Diseases, Faculty of Medicine, Ramathibodi Hospital, Mahidol University, Bangkok, Thailand; HIV-NAT, Thai Red Cross AIDS Research Centre, Bangkok, Thailand; Department of Global Health and Amsterdam Institute for Global Health and Development, Amsterdam University Medical Centers, University of Amsterdam, Amsterdam, The Netherlands; HIV-NAT, Thai Red Cross AIDS Research Centre, Bangkok, Thailand; Center of Excellence in Tuberculosis, Faculty of Medicine, Chulalongkorn University, Bangkok, Thailand; HIV-NAT, Thai Red Cross AIDS Research Centre, Bangkok, Thailand; HIV-NAT, Thai Red Cross AIDS Research Centre, Bangkok, Thailand; Department of Pathology, Faculty of Medicine, Ramathibodi Hospital, Mahidol University, Bangkok, Thailand; HIV-NAT, Thai Red Cross AIDS Research Centre, Bangkok, Thailand; Center of Excellence in Tuberculosis, Faculty of Medicine, Chulalongkorn University, Bangkok, Thailand; Biostatistics Excellence Centre, Faculty of Medicine, Chulalongkorn University, Bangkok, Thailand; The Kirby Institute, University of New South Wales, Sydney, Australia; Department of Health Sciences, Vrije Universiteit Amsterdam, Amsterdam Public Health Research Institute, Amsterdam, The Netherlands; Department of Global Health and Amsterdam Institute for Global Health and Development, Amsterdam University Medical Centers, University of Amsterdam, Amsterdam, The Netherlands

**Keywords:** CMV serology, CMV viremia, human cytomegalovirus, people with HIV, tuberculosis

## Abstract

**Background:**

Emerging evidence suggests that human cytomegalovirus (HCMV) infection is associated with tuberculosis (TB) disease. This study aimed to assess the relationship between the dynamics of HCMV markers and TB disease in people with human immunodeficiency virus (PWH).

**Methods:**

In a case-control study nested within a Thai HIV cohort, stored samples from people with HIV who were diagnosed with TB disease after antiretroviral therapy (ART) (cases) and TB-negative controls matched 1:2 on ART duration. HCMV DNA, immunoglobulin M (IgM), and immunoglobulin G (IgG) were measured at 3 timepoints: (*i*) 6–24 months before TB diagnosis (pre-TB visit), (*ii*) at TB diagnosis (TB visit), and (*iii*) 6–24 months after TB diagnosis (post-TB visit).

**Results:**

We enrolled 34 TB cases and 68 controls, the majority of whom were male (56% vs 60%). At the pre-TB visit, all participants were IgG seropositive, and there were no significant differences in the proportion with HCMV viremia or in HCMV IgM or IgG levels. At the TB diagnosis visit, the proportion of HCMV viremia was higher among TB cases compared with controls (34.5% vs 13.8%; *P* = .028). HCMV IgM geometric mean concentration (GMC) was higher in cases (0.17 vs 0.12 AU/mL; geometric mean ratio, 1.38 [95% confidence interval, 1.01–1.87]; *P* = .039). At the post-TB visit, HCMV viremia remained more frequent in cases (31.8% vs 4.5%; *P* = .006).

**Conclusions:**

HCMV viremia and serology measured 6–24 months before TB diagnosis in cases did not differ from those in matched controls. At TB diagnosis, people with HIV with TB had higher proportion of HCMV viremia and higher GMC of HCMV IgM, possibly reflecting HCMV reactivation due to TB disease.

Tuberculosis (TB) is one of the leading causes of death among people with human immunodeficiency virus (HIV). In 2023, TB caused an estimated 1.25 million deaths, including 161 000 among people with HIV (PWH) [1].

Although the introduction of antiretroviral therapy (ART) has substantially reduced the incidence of TB [2], ongoing TB risk remains high among PWH, despite long-term suppressive ART [3]. There is considerable interest in understanding the factors that contribute to the development of TB disease in this population.

Emerging evidence from epidemiological and immunological studies suggests that there is a relationship between human cytomegalovirus (HCMV) infection and TB disease [4–11]. A previous report highlighting epidemiological similarities between TB and HCMV infection hypothesized that HCMV infection may increase the risk of developing TB disease, possibly through early type I interferon (IFN) induction by infected myeloid and dendritic cells [4]. In human and murine models, excessive type I IFN induction is linked to an eicosanoid imbalance associated with TB disease exacerbation [12]. In addition, HCMV uses multiple immune evasion strategies that impair effective T-cell responses [13], including disrupted antigen presentation and secretion of immunomodulatory proteins, which could create conditions that favor mycobacterial proliferation [14].

A number of studies have explored the relationship between HCMV and adverse health outcomes, including incident TB. A case-control analysis among HIV-negative infants and adolescents reported that immune activation was associated with an increased risk of developing TB disease [5]. A previous retrospective study among Thai PWH showed that high HCMV viremia at ART initiation was associated increased risk of mortality [15]. High-level cytomegalovirus (CMV) viremia at baseline in individuals with advanced HIV and cryptococcal meningitis was associated with increased TB disease incidence and mortality during long-term follow-up in a prospective cohort of PWH in Uganda [16].

The temporal relationship of acquisition of HCMV and subsequent development of TB disease has been investigated in previous studies in which the majority of participants were HIV negative. A case-control study in healthy South African infants showed that HCMV-specific IFN-γ response measured at 4–6 months of age was a risk factor for the development of TB disease over the next 3 years of life, and this risk was greatest during the first 10 months of follow-up [8]. The magnitude of HCMV immunoglobulin G (IgG) was associated with increased risk of active TB disease up to 10 years before diagnosis in Ugandan adults [7]. These results raise the hypothesis that HCMV infection may play a role as a precipitating factor for TB disease. Gaining insight into the temporal relationship and interplay between these factors could lead to better understanding the drivers of TB disease. In addition, evidence regarding the dynamics of CMV infection in response to TB treatment remains limited. Furthermore, no studies have examined this hypothesis in PWH with long-term ART. In this study, we aimed to compare HCMV markers measured within 6–24 months before TB diagnosis using a matched nested case-control design.

## METHODS

### Study Populations and Setting

We conducted a matched nested case-control study within the HIV-NAT 006 (HN006) prospective, clinic-based cohort that has enrolled adults PWH aged ≥18 years since 1996 (ClinicalTrials.gov identifier NCT00411983). The HN006 study protocol was approved by the medical ethics committee of the Faculty of Medicine, Chulalongkorn University, Bangkok, Thailand (institutional review board No. 161/45) and executed in accordance with Good Clinical Practice guidelines and the principles of the Declaration of Helsinki [17]. All participants in the HN006 cohort provided written informed consent for the use of stored plasma samples in this study.

In this cohort, participants were seen every 6 months at HIV-NAT, Thai Red Cross AIDS Research Centre, Bangkok. Care for HIV infection was provided according to the Thai national treatment guidelines at the time when participants came for their follow-up visits. The following variables were collected at each clinic visit: body temperature, body mass index, physical examination findings, full differential blood counts, lipid profile, creatinine, and alanine aminotransferase. CD4^+^ and CD8^+^ lymphocyte counts performed by automated fluorescence-activated flow cytometry, as well as plasma HIV RNA levels, were done every 12 months. Plasma samples were collected and stored at each visit. Ten milliliters of whole blood was collected in ethylenediaminetetraacetic acid tubes and centrifuged (3000 rpm) at 4^ο^C for 20 minutes. Plasma samples were aliquoted into volumes of 500 μL and stored at ≤ −70^ο^C until analysis. The selected plasma samples were shipped to the Ramathibodi hospital for analysis.

The stored plasma samples from participants who were diagnosed with TB disease (cases) were assessed for HCMV DNA and HCMV immunoglobulin M (IgM) and IgG and compared with participants who were not diagnosed with TB disease (controls). We assessed the association between HCMV infection and TB at 3 timepoints: (*i*) 6–24 months prior to TB diagnosis in cases (pre-TB visit); (*ii*) at the time of TB diagnosis (TB visit) with an allowable window for sample collection of 2 months before or after initiation of TB treatment; and (*iii*) 6–24 months after TB diagnosis in cases (post-TB visit).

### TB Case and Control Definitions and Inclusion and Exclusion Criteria

During follow-up, participants with symptoms or signs suggestive of TB disease underwent evaluation, including physical examination, chest radiography, sputum smear microscopy, culture for *Mycobacterium tuberculosis*, and Xpert MTB/RIF testing (available from 2012 onward). TB disease was classified as bacteriologically confirmed if smear, culture (with drug susceptibility testing for all positive cultures), or Xpert results were positive, and as clinically diagnosed if radiologic findings were compatible with TB without bacteriological confirmation, together with a favorable response to anti-TB therapy.

TB cases in this study included PWH with symptomatic pulmonary TB who had stored samples from a pre-TB visit (6–24 months before diagnosis). Controls were selected from the HN006 cohort and matched 1:2 based on ART duration (±1 year from the case's diagnosis date). Clinical records confirmed that controls had no TB-related symptoms during follow-up. Only controls with stored samples from the corresponding time point of the matched case were eligible; if multiple candidates were available, 2 were randomly selected. Latent TB infection was not assessed because TB preventive therapy was not recommended in Thailand during the study period.

### Detection of HCMV Viremia

Quantitative HCMV DNA polymerase chain reaction (PCR) was performed in stored plasma samples of TB cases and controls at 3 different time points for the participants with available specimens. A commercial HCMV PCR kit (Alinity m CMV AMP Kit, Abbott, IL, USA) was used according to the manufacturer's recommendations. This assay utilizes real-time PCR to amplify and detect CMV genomic DNA sequences that have been extracted from the plasma specimens. The plasma specimens were thawed at 15°C to 30°C and processed immediately after thawing as undiluted specimens. This assay had a HCMV DNA limit of detection (LOD) of 30 IU/mL in plasma. HCMV viremia was defined as the detection of HCMV DNA in the plasma sample.

### Detection of HCMV IgG and IgM Antibodies

The IgG and CMV IgM antibodies to HCMV were detected by chemiluminescent microparticle immunoassay kits (Abbott Ireland Diagnostic Division). The collected specimens were processed immediately after thawing and mixed using a vortex mixer. They were performed with a fully automatic analyzer (Alinity i, Abbott). Individuals were considered to be seropositive if either the HCMV IgG or IgM was ≥6.0 AU/mL or ≥1.0 AU/mL, respectively.

### Statistical Analysis

Power calculations assumed the prevalence of HCMV viremia among controls would be 5% [15]. With 34 cases available and 2 controls enrolled for each case, the minimum detectable odds ratio with 80% power and a 2-sided significance level of 5% would be 3.19.

Binary variables were described as frequency (with percentages). For continuous variables, normally distributed and nonnormally distributed data were presented as mean with standard deviation and median with interquartile range (IQR), respectively. Cross-sectional comparisons of the proportion of TB cases and controls with detectable HCMV viremia were described at each study visit, and the HCMV concentrations in those with detectable plasma concentrations were described as median (IQR). The geometric mean concentrations (GMCs) and their 95% confidence interval (CIs) were calculated for HCMV IgM and IgG at each visit.

Formal comparisons of HCMV IgM and IgG between TB cases and controls were made using geometric mean ratio (GMR). Between-group comparisons of categorical variables were made with a χ^2^ or Fisher exact test as appropriate, and comparisons of continuous variables were made using a Mann–Whitney *U* test.

We also conducted a sensitivity analysis where the data for the pre-TB visit were limited to participants in whom the stored plasma sample was collected 6–12 months before TB diagnosis. Associations with *P* values <.05 were considered statistically significant. Statistical analyses were performed using Stata version 17.0 software (StataCorp, College Station, TX, USA).

## RESULTS

### Description of Study Cohort and Characteristics of TB Cases and Controls

**Figure 1.**
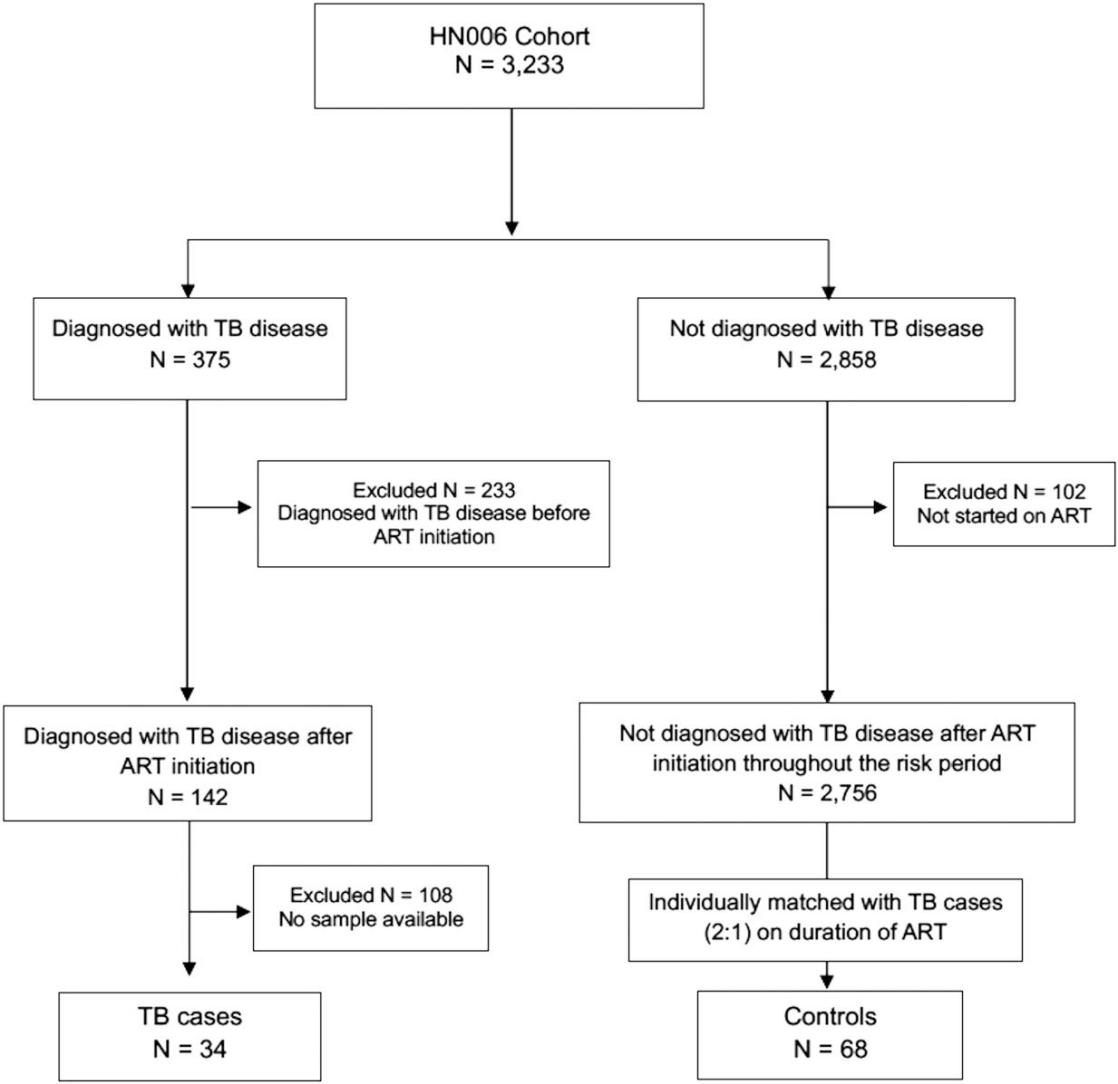
Flow diagram of tuberculosis cases and matched controls with stored samples. Abbreviations: ART, antiretroviral therapy; TB, tuberculosis.

Thirty-four TB cases and 68 control participants, individually matched by duration of ART (± 1 year), were enrolled (Figure 1). All participants were Thai and the majority were male (56% TB cases vs 60% controls). The median age at TB visit was 41.7 (IQR, 33.8–51.4) years and 44.3 (IQR, 37.5–52.2) years for TB cases and controls, respectively. The median duration of ART was 11.1 (IQR, 6.2–17.6) years in TB cases and 10.6 (IQR, 6.7–17.2) years in controls. The majority (97%) were receiving efavirenz-based ART. Median CD4 counts in controls were higher than TB cases (606 [IQR, 449–864] cells/μL vs 427 [IQR, 213–611] cells/μL; *P* < .001). The proportion of participants with detectable HIV viral load did not differ significantly between the 2 groups (8.8% TB cases vs 1.5% controls; *P* = .071). All of the TB cases had drug-susceptible pulmonary TB and were successfully treated with the standard TB 6-month regimen. Characteristics of TB and control participants are shown in Table 1.

**Table 1.** Characteristics of Tuberculosis (TB) Case and Control Participants at the Matched Visit (TB Visit)

Clinical Characteristics	TB Patients (n = 34)	Controls (n = 68)	*P* Value
Age, y, median (IQR)	41.7 (33.8–51.4)	44.3 (37.5–52.2)	.654
Male sex^a^, No. (%)	19 (55.8)	41 (60.3)	.670
BMI, kg/m^2^, median (IQR)	19.8 (17.7–21.6)	23.7 (21.7–26.4)	<.001
Hemoglobin level, g/dL, median (IQR)	12.6 (11.9–13.7)	14.45 (13.1- 15.2)	<.001
CD4 count, cells/μL, median (IQR)	427 (213–611)	606 (449–864)	<.001
Detectable HIV viral load, No. (%)	3 (8.8)	1 (1.5)	.071
Duration of ART, y, median (IQR)	11.1 (6.2–17.6)	10.6 (6.7–17.2)	.887

Abbreviations: ART, antiretroviral therapy; BMI, body mass index; HIV, human immunodeficiency virus; IQR, interquartile range; TB, tuberculosis.

^a^Refers to sex assigned at birth.

### HCMV Viremia in TB Cases and Controls at Each Visit

The median time from the pre-TB visit to TB diagnosis was 11.1 (IQR, 6.1–12.6) months. At the pre-TB visit, we observed no significant differences in the proportion with HCMV viremia between TB cases and controls (7/34 [21%] vs 12/68 [18%]; odds ratio, 1.20 [95% CI, .43–3.34]; *P* = .72) (Table 2). Among participants who had detectable HCMV, the median HCMV viral load was not significantly different, with the majority of both cases and controls having levels equivalent to the detection limit. None of the patients developed CMV end-organ disease.

**Table 2.** Human Cytomegalovirus Viremia and Viral Load in Participants by Visit

Parameter	Pre-TB Visit			TB Visit			Post-TB Visit		
	TB	Controls	*P* Value	TB	Controls	*P* Value	TB	Controls	*P* Value
HCMV viremia^a^, no./No. (%)	7/34 (20.5)	12/68 (17.6)	.719^b^	10/29 (34.5)	8/58 (13.8)	.028^b^	7/22 (31.8)	2/44 (4.5)	.006^c^
HCMV viral load when detectable^d^, IU/mL, median (IQR)	30 (30–33)	30 (30–30)	.21^e^	30 (30–251)	30 (30–30)	.07^e^	30 (30–30)	30 (30–30)	1.00^e^

Abbreviations: HCMV, human cytomegalovirus; IQR, interquartile range; TB, tuberculosis.

^a^Defined as the detection of HCMV DNA.

^b^χ^2^ test.

^c^Fisher exact test.

^d^Refers to HCMV DNA levels measurable at the assay's limit of detection of 30 IU/mL.

^e^Mann–Whitney *U* test.

**Figure 2.**
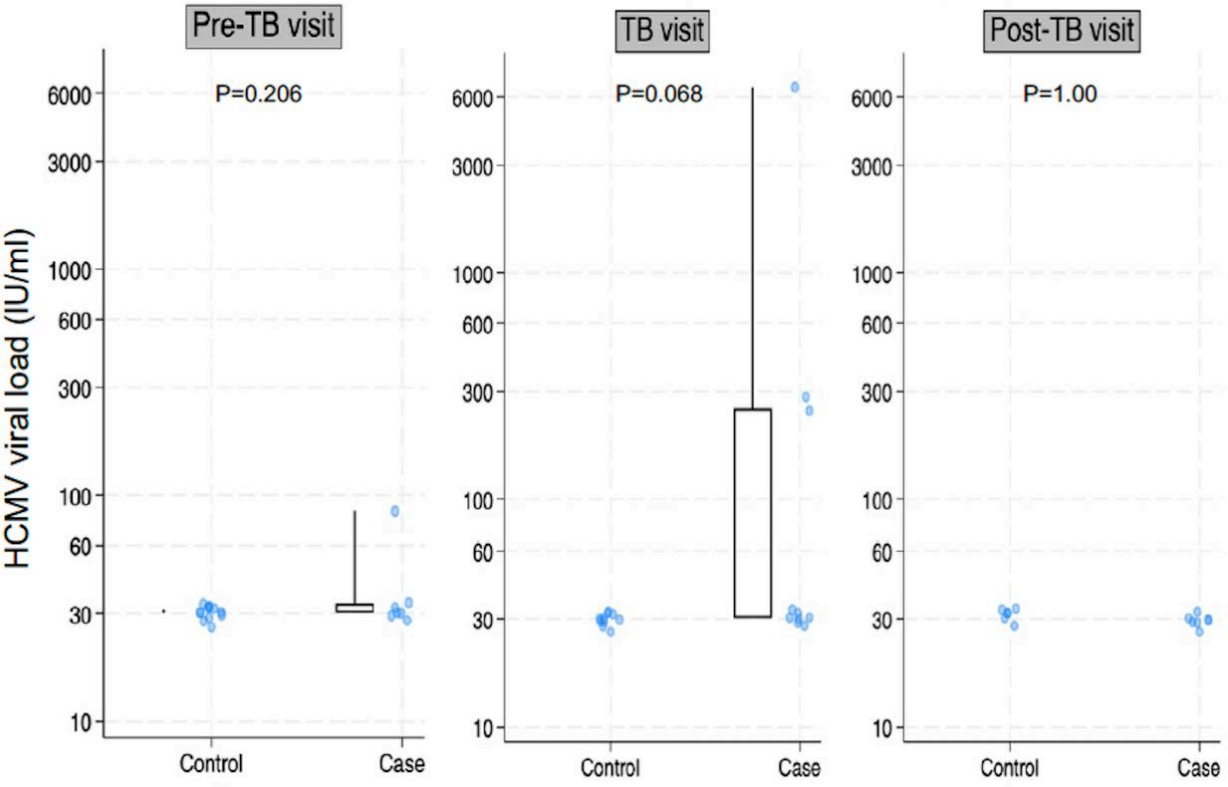
Human cytomegalovirus viral load among samples with detectable viremia, by visit and study group. The assay’s limit of detection is 30 IU/mL. Boxes represent median and interquartile range; whiskers represent maximum values. Abbreviations: HCMV, human cytomegalovirus; TB, tuberculosis.

At the TB diagnosis visit, the proportion of participants with HCMV viremia was significantly higher among PWH with TB than among controls at the corresponding visit (10/29 [34.5%] vs 8/58 [13.8%]; *P* = .028; Table 2). Median HCMV viral titer was 30 (IQR, 30–251; range, 30–6606) IU/mL in the cases versus 30 (IQR, 30–30; range, 30–30) IU/mL in the controls (*P* = .07; Figure 2). At the post-TB visit, we found a higher proportion with HCMV viremia in TB cases compared with controls (7/22 [31.8%] vs 2/44 [4.5%]; *P* = .006); there was no difference in the HCMV viral load at this timepoint.

In our sensitivity analysis where we restricted the time of sample collection to 6–12 months before TB diagnosis, we similarly found no significant differences in the proportion of study groups with HCMV viremia (3/22 [14%] vs 5/44 [11%] for TB cases and control, respectively; *P* = .54).

### HCMV IgM and IgG Antibody Levels Between TB Cases and Controls

All of participants in our study were HCMV IgG seropositive. At the pre-TB visit, we observed no significant differences in the HCMV IgM GMC (0.13 vs 0.12 AU/mL; *P* = .402) and HCMV IgG GMC (225.6 vs 206.6 AU/mL; *P* = .179) among TB cases and controls (Table 3).

**Table 3.** Human Cytomegalovirus Serology in Participants by Visit

Parameters	Pre-TB Visit				TB Visit				Post-TB Visit			
	TB	Controls	GMR (95% CI)	*P* Value^a^	TB	Controls	GMR (95% CI)	*P* Value^a^	TB	Controls	GMR (95% CI)	*P* Value^a^
HCMV IgM GMC (95% CI), AU/mL	0.13 (.10–.16)	0.12 (.10–.13)	1.11 (.86–1.44)	.402	0.17 (.12–.23)	0.12 (.10–.14)	1.38 (1.01–1.87)	.039	0.15 (.11–.21)	0.12 (.10–.15)	1.20 (.83–1.73)	.317
HCMV IgG GMC (95% CI), AU/mL	225.58 (210.16–242.12)	206.6 (190.15–224.62)	1.09 (.96–1.24)	.179	232.00 (216.64–248.45)	205.47 (188.04–224.50)	1.13 (.98–1.29)	.081	234.49 (218.37–251.81)	209.24 (193.00–226.85)	1.12 (.98–1.27)	.078

Abbreviations: CI, confidence interval; GMC, geometric mean concentration; GMR, geometric mean ratio; HCMV, human cytomegalovirus; IgG, immunoglobulin G; IgM, immunoglobulin M; TB, tuberculosis.

^a^ *t*-test.

At the TB diagnosis visit, the HCMV IgM GMC was higher in the TB cases than controls (0.17 vs 0.12 AU/mL; GMR, 1.38 [95% CI, 1.01–1.87]; *P* = .039). At the post-TB visit, we found no significant differences in the HCMV IgM and IgG GMC.

## DISCUSSION

In this matched nested case-control study among PWH with TB and controls who had been taking ART for a median duration >10 years, HCMV viremia and serology measured 6–24 months before TB diagnosis in cases did not differ from those in matched controls. At the time of TB diagnosis, PWH with TB had higher proportion of HCMV viremia and higher GMC of HCMV IgM compared with controls. At post-TB visit, PWH with TB exhibited a higher proportion of HCMV viremia, but not HCMV serology, compared with controls.

This is in contrast with the results from a longitudinal birth cohort in South African infants who were tested serially for active HCMV infection by PCR during the first year of life and followed for incident TB until the age of 9 years. In this latter study, infants who acquired HCMV before age 12 months had an increased risk of TB disease (adjusted hazard ratio, 3.2 [95% CI, 1.6–6.4]; *P* = .001), and those with a high CMV viral load seemed to be at highest risk [9].

There are a number of possible explanations which may explain the discordant findings between the South African cohort and our study. First, the African infants were HIV negative and acquired HCMV for the first time in their life. Primary HCMV infection elicits robust innate and adaptive immune responses [18]. The immunological alterations associated with HCMV infection may contribute to increased susceptibility to TB disease in young children [9]. In comparison, our study participants were adult PWH in Thailand, a country with high seroprevalence of HCMV in adults [19, 20], and all of our participants had been exposed to HCMV infection. The pattern of immune activation during HCMV reactivation differs substantially from that observed in primary infection. During reactivation, preexisting CMV-specific memory T cells are rapidly mobilized, and antibody-secreting plasma cells are promptly engaged, thus generating a prompt and focused immune reaction that restricts viral replication [18]. In our study, nearly all detectable PCR values represented viral loads at the LOD. Such low-level reactivation may not induce sufficient immunologic perturbation to affect the immune containment of *M tuberculosis*. In the South African infant cohort, by contrast, there was evidence of a dose-response relationship between HCMV load and TB risk [9].

Second, we used a different method to assess the HCMV infection. Our study examined HCMV viremia by quantitative PCR in plasma samples, while the African infant cohort assessed tested nasopharyngeal swabs for HCMV-specific DNA. A previous prospective birth cohort study in The Gambia showed higher rates of HCMV DNA detection in maternal oral secretions than in plasma samples [21]. Last, the timing of sample collection may also have impacted our results. The time period for the pre-TB visits in our study ranged from 6 to 24 months before TB diagnosis. The single sample collection during this long period may have been insufficient to capture HCMV viremia. Our sensitivity analysis restricted to participants for whom we had the sample collected <12 months before TB diagnosis also showed similar results. A previous study demonstrated a median time to HCMV viremia clearance of 13.5 (range, 5–40) weeks in PWH who started on ART without specific anti-HCMV therapy [22]; therefore, the long pre-TB visit window may have missed a period of HCMV viremia that happened in between.

In contrast to the pre-TB visit, we found a higher proportion of HCMV viremia and higher HCMV IgM level among cases with TB disease compared with controls at the time of the TB diagnosis visit. There are 2 possibilities regarding the increased HCMV viremia observed at this visit: (*i*) HCMV viremia persisted earlier, which contributed to the development of TB disease; or (*ii*) TB disease disrupts immune homeostasis and reinforces inflammatory responses, facilitating HCMV reactivation. TB is known to drive inflammation [23, 24] and evidence suggests the natural stimulus for HCMV reactivation is an inflammatory immune response to infection [25, 26]. However, the study design does not allow us to distinguish between these 2 explanations.

In our study, we found no association between the levels of HCMV IgG in serum samples taken before a TB diagnosis and the risk of developing TB disease. This contrasts with previous research in Ugandan adults [7], which assessed HCMV IgG titers from samples collected up to 10 years before TB diagnosis, representing a substantially broader sampling window than that used in our study. The difference in findings may reflect variations in study populations, highlighting the need for more research to better understand the relationship between HCMV, TB, and HIV coinfection.

There are several limitations in our study in addition to those already mentioned. Our study sample size was small, which may limit the precision of our results. In addition, the PCR assay employed in our study cannot differentiate reactivation of the same HCMV strain or reinfection with other strains. Due to the retrospective nature of the study, we were unable to assess other confounding factors that may influence the risk of HCMV reinfection, such as socioeconomic status. Last, the long interval between visits may be insufficient to capture the viremic period. Future studies that specifically investigate strain-specific HCMV infection with more frequent sampling would be helpful to identify the etiology of HCMV infection in relation to TB.

The strengths of our study include the longitudinal measurement of TB disease and CMV infection, allowing us to understand the temporal relationship of these diseases. In addition, this HIV cohort represents the longest longitudinal follow-up in Southeast Asia, with systematic data collection conducted under research conditions for >25 years.

In conclusion, our findings show that HCMV viremia and serology measured 6–24 months before TB diagnosis in cases did not differ from those in matched controls, while HCMV viremia and IgM responses were more common in cases at the TB diagnosis.

Considering the paucity of studies linking HCMV viral load or serology levels and TB disease, we believe it will be important to conduct longitudinal studies to further evaluate the association between HCMV and TB disease, especially in PWH in high-TB-burden settings.
